# Diet-Gut Microbiota Interactions and Gestational Diabetes Mellitus (GDM)

**DOI:** 10.3390/nu11020330

**Published:** 2019-02-03

**Authors:** Valentina Ponzo, Debora Fedele, Ilaria Goitre, Filomena Leone, Antonela Lezo, Clara Monzeglio, Concetta Finocchiaro, Ezio Ghigo, Simona Bo

**Affiliations:** 1 Department of Medical Sciences, University of Turin, 10126 Turin, Italy; valeponzo1@yahoo.it (V.P.); ilaria.goitre@libero.it (I.G.); ezio.ghigo@unito.it (E.G.); 2 Dietetic and Clinical Nutrition Unit, S. Giovanni Battista Hospital, Città della Salute e della Scienza, 10126 Turin, Italy; d.fedele85@gmail.com (D.F.); c.finocchiaro@cittadellasalute.to.it (C.F.); 3 Clinical Nutrition Unit, S. Anna Hospital, Città della Salute e della Scienza, 10126 Turin, Italy; filomena.leone@unito.it (F.L.); a.lezo@cittadellasalute.to.it (A.L.); 4 Gynecology and Obstetrics Unit, S. Anna Hospital, Città della Salute e della Scienza, 10126 Turin, Italy; c.monzeglio@cittadellasalute.to.it

**Keywords:** diet, gestational diabetes mellitus, microbiota, pregnancy

## Abstract

Medical nutritional therapy is the first-line approach in managing gestational diabetes mellitus (GDM). Diet is also a powerful modulator of the gut microbiota, whose impact on insulin resistance and the inflammatory response in the host are well known. Changes in the gut microbiota composition have been described in pregnancies either before the onset of GDM or after its diagnosis. The possible modulation of the gut microbiota by dietary interventions in pregnancy is a topic of emerging interest, in consideration of the potential effects on maternal and consequently neonatal health. To date, very few data from observational studies are available about the associations between diet and the gut microbiota in pregnancy complicated by GDM. In this review, we analyzed the available data and discussed the current knowledge about diet manipulation in order to shape the gut microbiota in pregnancy.

## 1. Introduction

Gestational diabetes mellitus (GDM) is an increasing public health concern that affects approximately 5–20% of pregnancies with rising prevalence [1,2]. It has been defined as any glucose intolerance with the first onset or recognition during pregnancy [3] and is associated with many adverse maternal and neonatal outcomes, such as preeclampsia, cesarean delivery, fetal macrosomia, shoulder dystocia, and neonatal hypoglycemia [4,5]. GDM is a transient state and glucose homeostasis is generally restored shortly after delivery. However, type 2 diabetes is expected to develop in 20–50% of these women within 10–20 years [6,7], and the GDM offspring shows a two- to eightfold increased risk of obesity, metabolic syndrome, type 2 diabetes, and impaired insulin secretion and sensitivity than offspring of women without GDM [8,9].

The appropriate management of maternal hyperglycemia can curtail both maternal and newborn morbidity [10], with medical nutrition therapy (MNT) being the first-line approach for the treatment of GDM [11]. In most cases, diet alone is sufficient to control blood glucose levels, however, up to half of the women fail to achieve a good metabolic control and require treatment with insulin or hypoglycemic drugs [12]. Despite the universally recognized importance of MNT in the treatment of GDM, there is no consensus about the optimal dietary macronutrient composition that best allows maternal euglycemia to be maintained.

The crucial role of the intestinal microbiota in modulating insulin resistance and the inflammatory response in GDM pregnancies has been reported by a few studies [13,14]. It is well known that diet is able to modulate the microbiota composition rapidly, within a few days [15,16]. Emerging evidence has shown a differential role of specific nutrients on metabolic outcomes, based on the individual microbial pattern. This is an intriguing and very important finding, suggesting that a food is not necessarily universally healthy per se but instead more suitable for specific individuals or conditions, thus strongly supporting a personalized approach to human nutrition. The potential impact of specific dietary interventions on the gut bacteria composition and function is of considerable interest in the search for the optimal strategy to prevent and treat GDM. At present, very few and controversial data are available.

Herein, we examine the updated evidence about this emerging topic. In particular, we analyze the changes in the gut microbiota and the diet–microbiota interactions occurring during healthy pregnancies and pregnancies complicated by GDM. Furthermore, we discuss the possible modulation of the gut microbiota composition by specific nutrients and foods, and the impact on the metabolic pattern of pregnant dysmetabolic women.

## 2. Methods

The following electronic databases were queried: PubMed (National Library of Medicine), the Cochrane Library, EMBASE, and Cumulative Index to Nursing and Allied Health Literature (CINAHL). The search strategy was performed using database specific subject headings and keywords; restrictions to human studies were placed. Hand searching the references of the studies and reviews on the field was employed to augment the search strategy.

## 3. Microbiota and Host Interactions

The term gut microbiota refers to all the microorganisms colonizing the human gastrointestinal tract [17]. Residential microbes have a symbiotic relationship with their host, since they are able to extract energy from foods that humans cannot digest, producing bioactive compounds, such as short-chain fatty acids (SCFAs) with proven beneficial effects on the host metabolism [18]. The gut microbiota can be considered a big virtual endocrine-metabolic organ, controlling many human pathways [19].

Dysbiosis, an altered microbiota composition, has been hypothesized to play a key role in the pathogenesis of many acute and chronic conditions, including metabolic diseases, such as obesity, insulin resistance, and both type 1 and type 2 diabetes mellitus (T2DM) [20,21]. Several mechanisms linking dysbiosis to dysmetabolic conditions have been identified, such as abnormal gut permeability, increasing absorption of lipopolysaccharide (LPS), abnormal SCFA production, aberrant conversion of primary bile acids to secondary bile acids, increased production of bacterial toxic substances, such as trimethylamine n-oxide (TMAO) [20,21]. These abnormalities lead to the activation of inflammatory and autoimmune pathways, autoantigen mimics, stimulation of the endocannabinoid system, aberrant gut peptide secretion, impairment of insulin signaling, increased energy extraction, and host fat storage [22,23].

## 4. Gut Microbiota Changes during Pregnancy

A variety of factors influence the gut microbiota composition, such as host genetic factors, comorbidities, antibiotic/prebiotic/probiotic use, dietary habits, and pregnancy [24].

**Figure 1:**
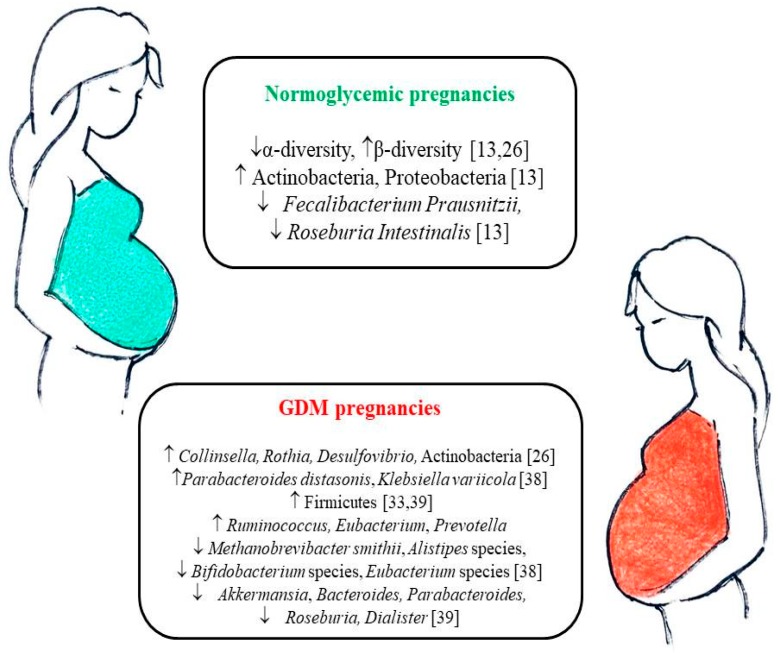
Gut microbiota changes during the course of normoglycemic pregnancies and GDM pregnancies. GDM: gestational diabetes mellitus. upward arrows mean an increased abundance, downward arrows mean a decreased abundance.

In healthy pregnancy, the gut microbiota composition undergoes dramatic alterations from the first to the third trimester [13,25,26]. Increased between-individual diversity (β-diversity) and decreased richness (intra-individual or α-diversity) have been reported, and a microbial pattern similar to that of non-pregnant adults with metabolic syndrome was found in late pregnancy (Figure 1) [13,26]. Indeed, profound hormonal, immunological, and metabolic changes take place above all during the third trimester to promote maternal weight gain, increasing circulating pro-inflammatory cytokines, and insulin resistance [14]. The reduced insulin sensitivity of late pregnancy is considered to be beneficial to support fetal growth and increased nutrient absorption [13,27]. The pregnancy variations in the gut microbial composition may contribute to all these metabolic changes [13,14].

However, at present, literature on microbiota composition in pregnancy is highly discordant, reporting no variations [28], increased Proteobacteria/Actinobacteria abundance, *Roseburia intestinalis* and *Faecalibacterium prausnitzii* reduction, and α-diversity decrease [13]. Gestational weight gain has been associated with higher concentrations of *Bacteroides* species [29], *Staphylococcus* [29,30], *Enterobacteriaceae* and *Escherichia coli* [30], reduced abundance of *Bifidobacterium* and *Akkermansia muciniphila* [30], and lower α-diversity [31]. Many correlations among specific taxa and gestational metabolic variables have been found, such as direct relationships between *Collinsella* and circulating levels of insulin, triglycerides, and very-low-density lipoproteins; *Sutterella* and C-reactive protein; Ruminococcaceae/Lachnospiraceae and leptin; Bacteroidaceae and ghrelin; *Coprococcus* and gastrointestinal polypeptide (GIP). Moreover, inverse relationships between *Blautia* and insulin values; *Faecalibacterium*/*Fusobacterium* ratios and blood glucose; *Odoribacter* and arterial blood pressure; Ruminococcaceae and GIP, and Prevotellaceae and ghrelin have been reported [27,32,33]. Therefore, gut microbiota might contribute to gestational metabolic changes through different mechanisms, although further studies on this topic are necessary.

## 5. Gut Microbiota in Pregnancies Complicated by GDM

The physiological insulin resistance of late-pregnancy is accentuated in women developing GDM, thus their insulin secretion is not sufficient to maintain euglycemia, leading to glucose intolerance [34]. Overall, insulin resistance has been found to be associated with a higher Firmicutes/Bacteroidetes ratio and a reduced abundance of butyrate-producing bacteria such as *Roseburia* and *Faecalibacterium prausnitzii* [35,36,37].

Koren et al., reported a reduced microbial richness in the first gestational trimester in women who later developed GDM, but no difference in the microbiota composition when compared to healthy pregnant women [13]. On the contrary, specific differences between GDM and normoglycemic women were reported by a few studies. Increased gut abundance of *Parabacteroides distasonis*, *Klebsiella variicola* [38], *Ruminococcus*, *Eubacterium*, *Prevotella* [39], *Collinsella*, *Rothia*, *Desulfovibrio*, Actinobacteria [26], Firmicutes [33,39] and reduced gut richness of *Methanobrevibacter smithii*, *Alistipes* species, *Bifidobacterium* species, *Eubacterium* species [38] *Akkermansia*, *Bacteroides*, *Parabacteroides*, *Roseburia*, and *Dialister* [39] were reported in GDM patients compared to normoglycemic controls (Figure 1). Some of the changes in the bacterial species related to GDM have been reported also in T2DM patients, such as the reduction in *Roseburia* and *Akkermansia muciniphila*, and the increase in Proteobacteria [21]. 

Functional analyses showed a greater abundance of membrane transport and energy metabolism pathways, lipopolysaccharide and phosphotransferase systems, and lower amino acid metabolic pathways in the microbiome of GDM patients [38].

If the changes in the gut microbiota contribute to or are a consequence of the development of GDM is a debated question. A different microbiota composition was found to precede the onset of GDM in early pregnancy, since both reduced microbial richness [13] and increased abundance of Ruminococcaceae family with a supposed subsequent increased energy harvest, pro-inflammatory status, and impaired insulin signaling, have been reported [40].

A few studies described a different microbiota within 3–16 months after delivery in women with a previous GDM (GDM+) compared to those with a previous normoglycemic pregnancy (GDM−). In particular, GDM+ women showed a higher abundance of Prevotellaceae, *Collinsella*, *Olsenella*, and *Clostridium* and a reduction in Firmicutes, *Fusobacterium* and the parent family *Fusobacteriaceae*, and genus *Ruminococcus* (*Ruminococcus* from Lachnospiraceae family) [26,41]. Indeed, no gut microbiota differences between GDM+ and GDM− women were found 5 years postpartum [42]. Therefore, the possibility that aberrant microbiota could contribute to the future development of T2DM in the GDM+ women becomes less probable.

Finally, the microbiota of GDM patients can be transmitted to the offspring, and colonization before birth by specific taxa associated with GDM occurs [32]. The finding of microbial inheritance during pregnancy underscores the importance of its early modulation.

## 6. Diet-Microbiota Interactions in Pregnancy

Diet is one of the most important factors that modulate the intestinal microbiota [16]. Foods influence the microbiota composition through direct and indirect mechanisms. Nutrients are able to interact directly with microbes by promoting or inhibiting their growth. On the other hand, dietary compounds affect the host metabolism and immune system and the subsequent changes shape the gut microbiota [43]. The effect of food nutrients on intestinal microbiota is rapid, and changes to the gut microbiota can occur within days after changing diet. However, the gut microbiota is considered to be relatively stable and generally reverts to its original status after short-term dietary changes [16]. Because of its resilience, early colonization of the infant gut by microbes sets the stage for the lifelong relatively stable adult microbiome, and pregnancy offers a window of opportunity for the microbiota shaping of the newborn.

Only a few studies have evaluated the role of nutrition on maternal gut microbiota during pregnancy. A lower fiber intake has been reported to be associated with reduced gut microbiota diversity and richness [44], greater abundance of *Collinsella*, a genus associated with T2DM [45,46,47], and greater abundance of *Sutterella*, a Proteobacteria with known pro-inflammatory capacity [48]. In pregnancy, vegetarian diets resulted in increased relative abundances of *Roseburia* and Lachnospiraceae, but no difference in α-diversity when compared to omnivorous diets [46]. A lower bacterial richness was found in pregnant women with high-fat, low-fiber intakes [44]. Furthermore, in overweight/obese women at early pregnancy stages, a high intake of saturated fatty acids (SFAs) was associated with the reduction of all indexes of microbiota richness [44], while, early after delivery, an increased SFA consumption was associated with reductions in Proteobacteria and Firmicutes abundance relative to the other phyla [48]. Monounsaturated fatty acids (MUFAs) has been associated with increase in the abundance of Firmicutes, Proteobacteria, and Bacteroidetes [48]. In pregnant women, fat-soluble vitamins seem to act as modulators of gut microbiota. Higher intakes of vitamin D were associated with reduced microbial α-diversity. The consumption of retinol and vitamin D was associated with a relative increase in abundance of the pro-inflammatory Proteobacteria phylum. On the contrary, vitamin E intake was associated with a relative decrease in abundance of Proteobacteria [48]. In overweight pregnant women, dietary fiber and n–3 polyunsaturated fatty acids (PUFAs) were associated with higher microbiota richness and lower serum zonulin levels [49], a protein that adversely modulates the permeability of gut tight junctions. Indeed, caution should be used in the interpretation of these results, since intakes were assessed by food frequency questionnaires [48] or food diaries [49], and, owing to the subjective nature of these data, associations between microbiota and micronutrients might be overestimated. 

In pregnancy, different nutrients were found to be related with gut microbiota diversity and composition. Owing to the emerging evidence of the potential role of human gut microbiota on metabolism and inflammation, future research is warranted in order to test the intriguing possibility that microbiota manipulation may improve maternal (and consequently neonatal) health.

## 7. Diet-Microbiota Interactions in GDM

The impact of diet on the microbiota of GDM patients was recently addressed by an observational study [33]. Forty-one overweight women with GDM diagnosed at 24–28 weeks of gestation received dietary recommendation from a registered dietitian, and their nutritional intakes and microbiota were evaluated after the diagnosis of GDM (enrolment) and at the 38th week of gestation. Women were considered as adherents to the given dietary recommendations if their intakes of rapidly absorbed sugars and fiber were respectively <10% of total energy and at least 20 g/day, and if they abolished alcohol intake at the end of the pregnancy. Adherents showed a significant decrease in *Bacteroides* [33], which is a genus that has been associated with high-fat animal-based diets [50]. Moreover, at enrolment, total fat intake was associated with higher abundance of *Alistipes* and protein intake with *Faecalibacterium* genus, while, at the end of the pregnancy, fiber intake was associated with the genus *Roseburia* [33]. However, none of these bacteria were associated with the changes in metabolic variables occurring during pregnancy in patients with GDM [33].

Therefore, the meaning of these associations remains unclear.

Gut microbes are able to use dietary methylamines (choline, l-carnitine, and phosphatidylcholine) and produce trimethylamine (TMA), which is further oxidized to TMAO by the hepatic enzyme flavin-containing monooxygenase 3 [51]. Red meat, eggs, dairy products, and salt-water fish are the main dietary sources of methylamines. Elevated circulating levels of TMAO have been associated with increased risk of T2DM and cardiovascular diseases [52]. Recently, in a large cross-sectional study, TMAO plasma concentrations in early and mid-pregnancy were positively related to increased odds of developing GDM [53]. In mice fed with a high-fat/high-sugar diet, TMAO promoted impaired glucose tolerance and adipose tissue inflammation [54]. At present, the pathogenetic role of plasma concentrations of TMAO on glucose metabolism is mostly unknown. Moreover, scarce information is available about the taxonomic composition of TMA-producing bacteria in humans, which are widely distributed across Firmicutes, Actinobacteria, and Proteobacteria phyla [55].

Further studies are needed on this important topic. Randomized controlled trials in which strategies of dietary manipulation during pregnancy are employed to shape the gut microbiota composition (such as reducing methylamines-contained foods, changing types of fiber intake) are strongly advisable in order to evaluate the possibility of GDM prevention or control.

## 8. Microbiota: A Novel Potential Therapeutic Target in GDM?

Despite the well-known role of MNT in the treatment of GDM [11], at present, there is no consensus about the optimal composition of the diet and about which nutritional scheme should be recommended [56,57]. Historically, the main focus of MNT in GDM was carbohydrate restriction, in particular simple sugar reduction [58], because this limitation reduces postprandial blood glucose levels, thus leading to a lower glucose exposure for the fetus and consequently a reduced risk for neonatal macrosomia [59]. Nevertheless, evidence of the benefits of carbohydrate restriction in GDM is scarce, and recently this strategy has been questioned due to the unintended consequences of the unavoidable increase in fat intake, if protein consumption remains in the recommended levels (15–20% total energy) [56,58]. It is well known that the amount and quality of fatty acids play relevant roles in the modulation of insulin resistance [60]. High-fat diets, in particular diets rich in SFAs, may promote insulin resistance through several mechanisms, such as insulin signaling interfering [60], the promotion of inflammation through tumor necrosis factor alpha (TNF-α) production [61], and increased oxidative stress [62].

We do not eat alone, but also feed our microbiota, therefore we should also consider the effects of food and nutrients on the composition of our intestinal microorganisms. From a “microbiota point of view”, the high fat intake could potentially increase pro-inflammatory bacteria, resulting in enhanced insulin resistance. High-fat diet has been associated with increased intestinal permeability caused by high LPS serum levels, endotoxemia, and low-grade inflammation [63,64]. In animal studies, the consumption of high-SFA diets was observed to reduce the phylum Bacteroidetes (in particular, *Bacteroides* and *Prevotella* genera), and the species *Lactobacillus* and *Bifidobacterium* [65]. In humans, high-fat diets have been reported to increase total anaerobic microbes, in particular the *Bacteroides* genus, while low-fat diets led to an increase in the abundance of *Bifidobacterium* concomitant with fasting glucose and total cholesterol reductions [15,66]. In human pregnancy, a high-fat diet determines an unfavorable microbial pattern, with a reduced bacterial richness, as described above [44]. Furthermore, the association between GDM and SFA intake has been documented [67].

A decade ago, doubts about the effectiveness of low-carbohydrate diets in GDM began to emerge and nowadays the focus has moved from carbohydrate restriction to carbohydrate quality; in particular, diets with low glycemic index (GI) and/or rich in complex unrefined carbohydrates have been studied with promising results [68,69,70]. Different types of complex carbohydrates exert different impacts on microbiota composition. First of all, the terminology used needs to be clarified. The generic term “fiber” (i.e., undigestible carbohydrates) in the context of microbiota is misleading. Not all fibers can be used by gut microbes, such as cellulose, while other carbohydrates that are not fibers can be fermented by our intestinal microorganisms, such as resistant starches. Recently, the term microbiota accessible carbohydrates (MACs) has been proposed [71]. Microbial fermentation of MACs produces SCFAs (acetate, propionate, and butyrate), which are able to activate G-protein-coupled receptors (GPRs), such as GPR41 and GPR43, triggering the secretion of gut hormones (glucagon-like peptide 1 -GLP-1, and peptide YY -PYY) by intestinal epithelial L-cells, and the secretion of leptin by adipocytes [18], resulting in appetite decrease [72,73], and improved insulin sensitivity [18]. SCFAs can suppress lipolysis, and the release of inflammatory mediators, such as nitric oxide (NO), TNF-α, interleukin 1-beta (IL-1β), and interleukin 6 (IL-6) [73,74,75,76,77]. Butyrate and propionate are generally considered beneficial [18], while the role of acetate is still controversial in humans [78]. Indeed, the role of SCFAs in energy homeostasis is ambiguous. Fecal SCFAs have been found to be increased in the gut microbiota of people with obesity [79] and this might indicate a greater ability to extract energy from undigested foods contributing to the development of obesity [18,80].

The intakes of different MACs were associated with different microbe growth: prebiotic fibers, such as fructans, polydextrose, fructooligosaccharides (FOS), and galactooligosaccharides (GOS) were linked to the growth of intestinal *Bifidobacteri* and *Lactobacilli* [81]; resistant starch was reported to increase the abundance of *Ruminococcus*, *E. rectale*, and *Roseburia* [15,82]. In animal studies, low-MAC diets resulted in over-grazing the mucus layer by the gut microbiota, potentially compromising the integrity of the mucosal barrier and increasing inflammation and susceptibility to pathogens [83]. Intriguingly, the selective use of specific MACs could have a different impact on microbiota composition and consequently on host metabolism. Therefore, not only carbohydrate quality but also the type of MAC should be considered in the definition of the optimal diet for GDM patients.

## 9. Towards Personalized Nutrition for GDM

The “one-size-fits-all” diet approach has been called into question for most metabolic disorders. The individual response to diets is influenced by genetic, epigenetic, and microbial influences [84]. The metabolic response to specific foods based on the individual gut microbiota composition is one of the emerging topics of interest in the field of personalized nutrition [85]. Recent studies have identified individual enterotypes with different responses to specific diets [86]. Based on the predominant genus, people can be divided into three different clusters, called “enterotypes”: *Bacteroides*, *Prevotella*, and *Ruminococcus* [87], with a high predominance of the first two enterotypes around the world. These clusters appear to be independent from nationality, sex, age, and body mass index (BMI), but substantially determined by dietary habits [88]. *Prevotella* (P-type) enterotype was associated with a plant-based diet, rich in carbohydrates, resistant starch, and fibers, whereas *Bacteroides* (B-type) enterotype was associated with high-fat, low-fiber Western diets [88,89,90]. Due to the specific microbial enzymatic capacity, the P-type would have a greater ability to produce propionate from fiber fermentation (in particular, arabinoxylans and β-glucans), with increased production of the satiating PYY and GLP-1 hormones [86]. The P-type subjects have been proven to lose weight with a high-fiber diet rich in arabinoxylans and β-glucans [91,92,93], while the B-type individuals do not benefit from these fibers. In a recent study, 3 days of barley kernel-based bread supplementation improved the glucose metabolism only in subjects with higher *Prevotella/Bacteroides* ratios [93], confirming the role of arabinoxylans and β-glucans in the P-type individuals. On the other hand, the B-type individuals could lose weight and improve their glucose metabolism with bifidobacteria-increasing interventions, including prebiotics, such as inulin and oligosaccharides, or symbiotic treatment [86].

A Prevotellaceae-dominated intestinal microbiome has been observed after 3–16 months from delivery in women with previous GDM when compared to women after a normoglycemic pregnancy [41]. Other studies confirmed an increased abundance of the genus *Prevotella* in GDM in the second [38] and third [39] trimester of gestation compared to normoglycemic subjects. Therefore, overall, in GDM patients it could be hypothesized that there is a beneficial effect resulting from the increased consumption of foods containing arabinoxylans and β-glucans, such as rye, wheat, barley, oats, sorghum, maize, millet, psyllium, and flaxseed. In GDM patients, *Bacteroides* abundance has been found to be either increased [32] or decreased [39]. A reduced abundance of *Bacteroides* at the end of pregnancy has been described in women with GDM with a higher intake of total fiber and oligosaccharides [33]. This reduction in *Bacteroides* abundance could be considered positive, as this genus has been associated with greater gestational weight gain [29]. Therefore, only in the subgroup of GDM patients with a higher relative abundance of *Bacteroides*, bifidogenic prebiotic fibers (FOS, GOS and inulin, contained in foods such as legumes, chicory roots, Jerusalem artichokes, onions, garlic, asparagus, daikon, and leek) might be beneficial to control the gestational weight gain.

A step towards personalized recommendations based on microbiota composition in a clinical setting was the recent production of a tool that is able to predict the individual impact of foods on postprandial glycemic responses based on an algorithm integrating clinical variables and gut microbiota composition [94]. Furthermore, lower glycemic response after eating different types of bread (artisanal sourdough bread and industrially made white bread) can be predicted in each person based only on microbiome data [95]. This suggests once again that giving the same dietary recommendations to all people has limited efficacy.

## 10. Limitations and Future Perspectives

The key points of this review are reported in Table 1. However, the limitations of the current knowledge should be recognized. At present, we are still far from finding a personalized dietary treatment for GDM patients. First of all, our knowledge about gut microbiota and diet response in pregnancy complicated by GDM is limited. Furthermore, gut microbiota is just one of the components that can determine personalized responses to diet therapy; future studies on personalized nutrition should take into account and integrate all the possible variables, including genetics and clinical parameters. Finally, doubts have recently been raised about the benefit of probiotics in preventing GDM, thus questioning the role of microbiota in pregnancy hyperglycemia [96].

Nevertheless, in addition to the traditional recommendations of consuming an adequate caloric intake, and reducing simple sugars and SFAs, the recommendation to increase the intake of specific type of fibers could be given. In particular, if there are no improvements in weight and metabolic outcomes, it could be beneficial to vary the type of MACs, increasing arabinoxylans and β-glucans rather than FOS and GOS.

## 11. Conclusions

The search for the optimal nutritional strategy in GDM patients remains an unresolved issue. In the study of the optimal nutritional scheme for GDM women, it is important that the potential benefits of diet for the mother, the fetus, and the maternal microbiota, which in turn will impact on the newborn microbiota, are taken into consideration.

## Figures and Tables

**Table 1:** Key points about diet-microbiota interactions in patients with GDM (gestational diabetes mellitus).

	Clinical Impact	Future Perspectives	Limitations
An impaired gut microbiota has been found in pregnancies complicated by GDM.	The microbiota of GDM patients can be transmitted to the offspring.	Early modulation of the gut microbiota might be warranted in women at risk of developing GDM.	Few, contrasting data available. Uncertainty about the causal relationship between gut dysbiosis and GDM.
Diet can shape the gut microbiota and the microbiota can use nutrients to produce bioactive compounds.	The gut microbiota rapidly changes with dietary modifications. However, it generally reverts to the original status with short-term dietary changes.	Long-term dietary manipulation during early pregnancy (or before pregnancy) to shape the gut microbiota composition might be a potential strategy for the prevention or control of GDM.	Limited data available. Randomized controlled trials are lacking.
The metabolic response to specific foods is based on the individual gut microbiota composition.	Weight change or glycemic responses to fiber-containing foods vary according to the predominant individual microbial pattern.	The recommended type of fiber could be individualized in GDM patients on the basis of the specific gut microbiota composition in order to obtain better metabolic outcomes.	Limited data available. Randomized controlled trials are lacking.
